# *Panax ginseng* Pharmacopuncture: Current Status of the Research and Future Challenges

**DOI:** 10.3390/biom10010033

**Published:** 2019-12-25

**Authors:** In-Seon Lee, Ki Sung Kang, Song-Yi Kim

**Affiliations:** 1 Acupuncture and Meridian Science Research Center, College of Korean Medicine, Kyung Hee University, Seoul 02453, Korea; islee4u@gmail.com; 2 College of Korean Medicine, Gachon University, Seongnam 13120, Korea

**Keywords:** *Panax ginseng*, ginsenoside, wild ginseng, pharmacopuncture, safety, clinical trials

## Abstract

Despite the increasing use of ginseng pharmacopuncture in clinical practice, evidence of its physiological effects, safety, and clinical outcomes is insufficient. The purpose of this review is to summarize previous studies and suggest future challenges for the clinical use of ginseng pharmacopuncture. We systematically searched clinical and animal studies that applied ginseng pharmacopuncture and reviewed the manufacturing processes of ginseng pharmacopuncture solution, safety, physiological responses, and clinical effects. Intravenous or point injection of the ginseng pharmacopuncture solution made by distillation extraction has been commonly used in studies. Ginseng pharmacopuncture does not show any toxicity in animals and humans, while it influenced the heart rate variability, pulse wave velocity, and protein synthesis in human subjects. In 25 case reports, patients with cancer, amyotrophic lateral sclerosis, skin wrinkles, and allergic rhinitis showed significant improvement of clinical outcomes. We found that more evidence is necessary to conclude that ginseng pharmacopuncture is safe and effective. First, the pharmacopuncture manufacturing process should be standardized on the basis of the safety and efficacy tests. Moreover, studies on the quantitative quality of the components of the solution and on the clinical comparison of various injection methods are required to improve clinical outcomes in the future.

## 1. Introduction

The root of *Panax ginseng C. A. Meyer* (*P. ginseng*) has been widely used as a tonic in East Asian countries such as Korea, China, and Japan since ancient times to improve organ functions and increase vital energy based on traditional medicine theory and clinical experiences [1]. According to the Qi and flavor theories, the theoretical pharmacology systems in traditional Asian medicine using the property and flavor of a medicine, it is slightly warm and nourishes the Qi and Yang. Therefore, it has been used to treat the Qi deficiency state in various diseases such as chronic illness, fatigue, and weak functions or organs [2]. The major active components of *P. ginseng* are ginsenosides (also called saponins, Table 1), and their mechanisms have been primarily investigated to reveal the pharmacological effects of *P. ginseng*. Ginsenosides are classified as protopanaxadiol (e.g., ginsenoside Rb1, Table 2) or protopanaxatriol (e.g., ginsenoside Rg1, Table 3). In addition to the ginsenosides, there are essential oil components, phenol compounds, polysaccharides, alkaloids, and nitrogen compounds (Table 1). Traditionally, wild ginseng has been regarded as being more effective than cultivated ginseng. Wild ginseng contains a higher level of ginsenoside Rb1 and Rg1 than cultivated ginseng [3], and proteomic analysis revealed that wild ginseng contains higher levels of amino acids, amino acid-related enzymes and proteins, and derivatives than cultivated ginseng [4].

Traditionally, *P. ginseng* is used in the decoction form and is decocted with boiling water for a certain number of hours and prepared as a drink [5]. More recently, ginseng concentrates, extracted using water or alcohol, are also used [6]. In addition, ginseng has been transformed into red ginseng or fermented black ginseng through steaming, repeated heating and drying, or fermentation [6,7]. In addition to oral intake of ginseng, whose clinical effects and safety have been demonstrated [8,9], ginseng has been also administered via injection (intravenous or intramuscular on either acupoints or non-acupoints), and this technique is called pharmacopuncture.

Pharmacopuncture is a relatively new acupuncture therapy in Traditional Korean Medicine (TKM) that combines acupuncture with herbal medicine [10]. Pharmacopuncture involves injection of filtered and sterilized herbal medicine extracts, which are extracted using different techniques (e.g., alcohol immersion, distillation, or pressing) depending on the herbs [11]. Thus, it simultaneously induces mechanical stimulation of acupoints and a pharmacological effect. It was originally developed under the name of ‘Aqua-acupuncture’ in the 1950s in China [10]. Aqua-acupuncture uses both herbal and non-herbal medicines and has been regarded as a combined therapy with traditional and Western medicine. However, pharmacopuncture in South Korea is exclusively associated with TKM, as it uses herbs that have been used in the form of other formulations (decoction, granule, etc.) and involves injection of the herbal extracts intravenously or into acupuncture points (acupoint), trigger points, or response points. The herbs and acupoints (in the case of herbal extracts injected into acupoints) are selected according to the meridian, Qi, and flavor theories, and the syndrome differentiation diagnosis protocol from TKM [2,11].

Ginseng pharmacopuncture is a typical single-herb pharmacopuncture, which contains diverse substances such as ginsenoside Rg1, ginsenoside Rb2, and phenolic compounds, and the amount of substances vary depending on the extraction methods (e.g., distilled versus ethanol extract [12]). A review of animal studies suggested that ginseng pharmacopuncture is useful in the prevention of diseases and strengthening immune response, especially in Yang insufficiency animal models induced by hydrocortisone acetate injection [13]. Furthermore, several studies have investigated the toxicity of ginseng pharmacopuncture, and its safety has been studied in animals [14,15]. On the basis of these lines of evidence, single-herb ginseng pharmacopuncture has been widely used in clinics as one of the standard TKM therapies in South Korea, while pharmacopuncture with several herbs combined (combination of various herbal extracts; e.g., Shenmai or Shenfu injections) has been more extensively used in China. Despite its wide usage in clinics, evidence is insufficient to prove whether ginseng pharmacopuncture therapy significantly improves the clinical outcomes and risk of adverse events (AEs) in patients.

In this study, we restrictively defined ginseng as the root of *Panax ginseng C. A. Meyer*, and our aim was to provide a comprehensive review of the clinical application of *P. ginseng* pharmacopuncture, which we referred to as ‘ginseng pharmacopuncture’, including its safety, physiological, and clinical responses. To achieve this goal, we (1) provided an overview of the physiological responses to and side effects of ginseng pharmacopuncture in animals or humans (patients and healthy participants), and (2) systematically reviewed previous clinical trials using ginseng pharmacopuncture in patients with various diseases. Lastly, we emphasized that more basic and clinical studies are needed to confirm the effects and safety of ginseng pharmacopuncture, and suggested future directions for developing ginseng pharmacopuncture as a safe and effective treatment for patients seeking TKM therapies.

## 2. Materials and Methods

### 2.1. Search Strategy

We used a systematic search strategy following the preferred reporting items for systematic reviews and meta-analyses (PRISMA) guidelines for systematic reviews. Electronic searches of articles were conducted from their inception to January 2018, without language restrictions in PubMed, the Research Information Sharing Service (RISS), the Korean Studies Information Service System (KISS), KoreaMed, and the China National Knowledge Infrastructure (CNKI). The search terms used were “ginseng” including *Panax ginseng* Radix, *Panax ginseng*, ginseng radix, or red ginseng; and “pharmacopuncture”, including aqua acupuncture, herbal/herb acupuncture, point-injection, or yakchim. “Red ginseng”, which is the most common processed type of *P. ginseng*, was added as a search term. Furthermore, a secondary search was performed by screening the reference lists of articles that met the inclusion criteria.

### 2.2. Study Selection, Data Extraction, and Data Analysis

Three categories of studies were included in the search strategy if they evaluated pharmacopuncture using *Panax ginseng C. A. Meyer* roots as follows: (1) preclinical trial in animals, (2) study of physiological responses in human subjects, and (3) clinical trial in patients to identify the clinical effects of ginseng pharmacopuncture.

We followed the process of systematic literature review. First, searched articles were screened on the basis of the title and abstract before the full text was assessed. Second, articles identified as duplicates or non-original studies such as reviews, opinions, or protocols were removed. Third, publications that did not meet our definition of ginseng pharmacopuncture (single-herb ginseng administered via intravenous or intramuscular injection), studies that used pharmacopuncture consisting multiple herbs, and articles not written in English, Chinese, or Korean were excluded. The included studies were classified as animal studies, studies on the physiological responses of healthy participants, or clinical trials in patients. After the selection of the articles to be analyzed, we extracted data from the included clinical trials by using a predefined form that contained the following items presented in a separate table: manufacturing process of pharmacopuncture solution and characteristics of participants (e.g., age, sex, disorder or symptoms), intervention details (injection site, volume of injection, number and duration of treatment and co-intervention), and clinical outcomes. Results of animal studies and physiological responses are summarized narratively in the manuscript.

## 3. Results

### 3.1. The Manufacturing Process of the Ginseng Pharmacopuncture Solution

**Figure 1:**
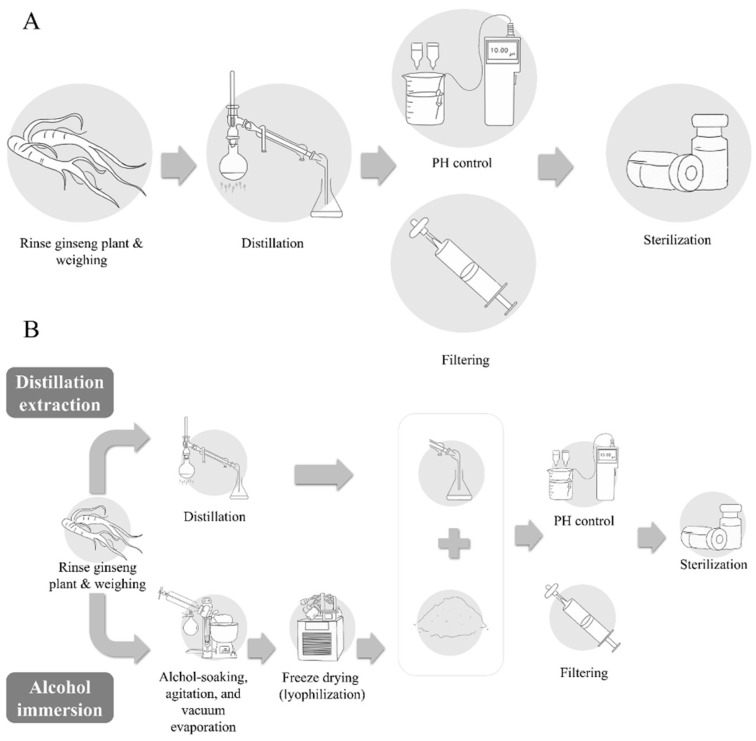
Manufacturing process of pharmacopuncture. (**A**) Distillation extraction; (**B**) combined method of distillation and alcohol immersion.

Distillation extraction, alcohol immersion, the combined method of distillation and alcohol immersion, compression, and dilution methods are used for the manufacture of pharmacopuncture solution [2,11]. Among these methods, the ginseng pharmacopuncture solution is generally produced using the distillation method. In the included studies, ginseng plant was rinsed with clean water (in some cases, the rhizome part of the ginseng is removed) followed by distillation and/or decoction, filtering, and sterilization of the solution. Rarely, in some cases, extracts of certain active or target substances such as polysaccharides from ginseng were used to make the solution [16] (Figure 1). Recently, the Accreditation System of External Herbal Dispensaries (EHDs) was announced. The quality of pharmacopuncture should be managed according to the EHDs and clinicians and patients can verify whether the pharmacopuncture solution is produced in herbal dispensaries with an EHD certification mark [17].

### 3.2. Physiological Response to Ginseng Pharmacopuncture in Human Subjects

In this review, 13 studies measured various physiological responses before and after receiving ginseng pharmacopuncture. In the healthy subjects, intravenous injection of wild ginseng pharmacopuncture did not change the blood pressure, pulse, temperature, respiration, and blood test indexes [18,19]. Wild ginseng pharmacopuncture injected at acupoints (ST36 and ST37) did not significantly change the acupuncture sensations as compared with other types of complex-herb pharmacopuncture (e.g., BUM pharmacopuncture made of *Calculus Bovis*, *Fel Ursi*, *Moschus*) and saline injection [20]. Another study showed that only the volume of injected solution, but not the stimulation methods (perpendicular versus transverse injection) at acupoints, affected the subjective intensity reporting of pharmacopuncture stimulation [21].

By contrast, randomized controlled trials (RCTs) showed that the sympathetic nervous system in healthy participants was activated significantly by wild ginseng pharmacopuncture injected at acupoints as compared with saline injection [22,23,24,25]. Intravenous wild ginseng pharmacopuncture decreased the mean heart rate variability (HRV) and pulse wave velocity (PWV) and increased the mean and standard deviation of normal R-R intervals and very low frequency oscillation power in patients with breast cancer [26], while it decreased the mean HRV and PWV and increased the mean normal R-R intervals in healthy volunteers [27]. Intravenous wild ginseng pharmacopuncture injection also affected pulse-related factors (e.g., increased the stability of the pulse wave) [28]. It also increased the level of proteins CR2-C3d, Ral-A, proapolipoprotein, apolipoprotein, transferrin, human hemoglobin, and vitamin D binding protein and reduced the transthyretin and antitrypsin levels in blood samples [29,30].

Current evidence suggests that ginseng pharmacopuncture may activate the sympathetic nervous system and change the protein synthesis mechanisms, while the sensory stimulation aspect (e.g., De-Qi sensation induced by needle injection at acupuncture points) may not specifically be involved in the effect of ginseng pharmacopuncture. However, the underlying mechanisms and physiological actions of ginseng pharmacopuncture have not yet been fully explained owing to insufficient evidence.

### 3.3. Safety Tests of Ginseng Pharmacopuncture

Several studies have tested the safety of ginseng pharmacopuncture in animals using different dosages and treatment frequencies. For example, both single administration (20 or 10 mL/kg) and repeated administration (10, 5 or 2.5 mL/kg for 4 weeks, once a day) of ginseng pharmacopuncture evoked no significant toxic responses in Sprague-Dawley rats (e.g., changes in mortality, histological observations, body weight, clinical signs, and food consumption behavior) [14,15]. In addition, pharmacopuncture using intravenous or intramuscular injection of radix ginseng (dried root of *P. ginseng*) at a single dose (0.1 0.5, or 1.0 mL/animal) did not change body weight, general condition, and hematological or biochemistry test results in rats [31,32].

In a case report, drug-induced liver injury (increased alkaline phosphatase level, white blood cells, and platelets; all indexes were outside the normal range) was suspected after intravenous wild ginseng pharmacopuncture treatment in a patient with shoulder pain, however, the causal influence of the wild ginseng pharmacopuncture treatment on the liver injury was not proven in the study [33]. In the RCT that compared the combination therapy of ginseng polysaccharides pharmacopuncture, Bupleurum pharmacopuncture, and paroxetine (pharmacopuncture group) with paroxetine monotherapy (control group), seven cases of AEs such as headache, dry mouth, or tremor were reported in the pharmacopuncture group. However, 21 cases of AEs such as diarrhea, constipation, weakness, or anorexia were reported in the control group, including nine cases of the same type of AEs as the treatment group [16].

In summary, the safety of ginseng pharmacopuncture injection has been mainly studied in animals, but more empirical investigations are necessary to prove its safety in humans.

### 3.4. Systematic Review of Clinical Studies

#### 3.4.1. Search Results

**Figure 2:**
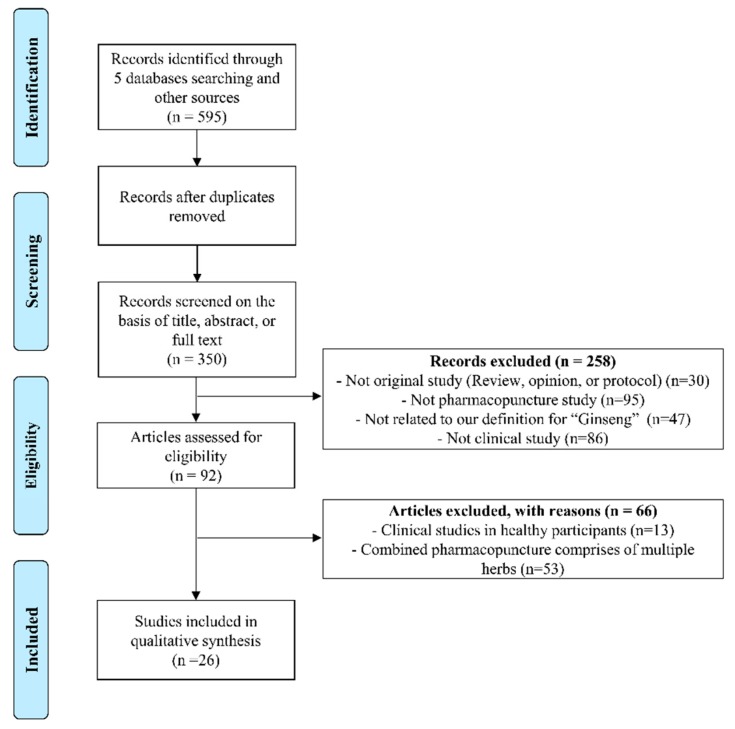
PRISMA flow chart for the search and selection of the included clinical trials.

Figure 2 summarizes the results of the literature search process for analysis of clinical studies. Our research strategy retrieved a total of 595 articles, 245 of which were duplicates. An additional 324 studies were discarded after screening the full text or abstract including 95 not related to pharmacopuncture, 47 not related to *P. ginseng*, 53 that used pharmacopuncture with combined herbs. Finally, 25 case reports (24 studies written in English [14,34,35,36,37,38,39,40,41,42,43,44,45,46,47,48,49,50,51,52,53,54,55] and one study in Chinese [56]) and one RCT (written in Chinese) [16] met the inclusion criteria and were incorporated in the systematic review (Figure 2 and Table 4).

#### 3.4.2. Participants and Settings

Twenty-four studies were conducted in Korea, and two studies were conducted in China [16,56]. Among the case reports, 18 used wild ginseng pharmacopuncture in patients with cancer [lung cancer (*n* = 17, patients); hepatocellular carcinoma, prostate cancer, colorectal cancer, or pancreatic cancer (*n* = 2, respectively); cervical cancer, thymus cancer, breast cancer, Signet ring cell carcinoma, bronchioloalveolar carcinoma, or tubulovillous adenoma (*n* = 1, respectively)] [34,35,36,37,38,39,40,41,42,43,44,45,46,47,48,49,50,51]. One hundred patients with rhinitis [56], 23 with skin wrinkles [21], three with amyotrophic lateral sclerosis (ALS) [53], and one with Behçet’s disease, hepatitis, hepatocirrhosis [52], cervical dysplasia [54], neurofibroma [14], and acute demyelinating encephalomyelitis [55] were included in the systematic review. A RCT included 102 patients with depression and compared the effect and AEs of combined therapy composed of ginseng polysaccharides pharmacopuncture, Bupleurum pharmacopuncture, and paroxetine with those of paroxetine-only treatment [16].

#### 3.4.3. Ginseng Pharmacopuncture Interventions

Among the 26 included studies, seven used wild ginseng pharmacopuncture [14,35,40,47,48,49,51], one study used ginseng polysaccharides pharmacopuncture [16], and 18 used cultivated ginseng pharmacopuncture.

**Figure 3:**
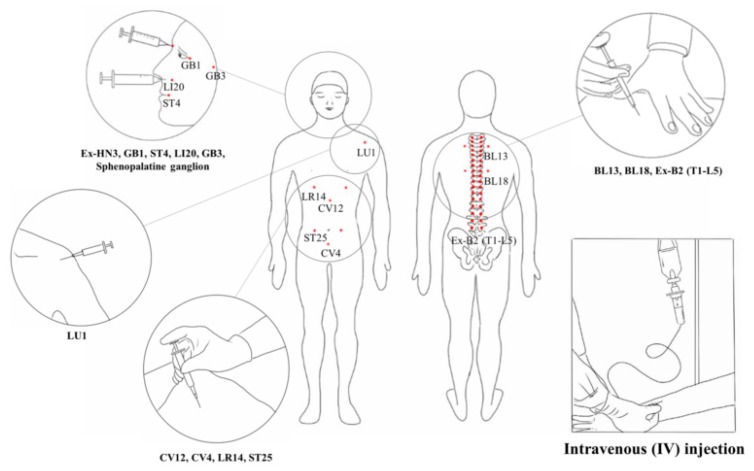
Intravenous and acupoint injections of pharmacopuncture. The area inside the circle means the acupoint injection site or each acupuncture point.

Four studies [21,39,48,56] applied ginseng pharmacopuncture alone, and the others used various co-interventions such as antidepressants, anticancer drugs, analgesics, herbal medication, chemotherapy, cupping, moxibustion, acupuncture, diuretics, rehabilitation, surgery, and pharmacopuncture using other herbs. Fifteen studies involved pharmacopuncture performed intravenously, 10 involved injections in acupoints (three studies used both intravenous and acupoint injections [34,37,40]), and one involved injection into the sphenopalatine ganglion [56] (Figure 3). Injection methods were not clearly described in one study [46].

#### 3.4.4. Outcome Measures

Clinical outcomes were classified into three categories as follows: objective measurement (e.g., tumor size in patients with cancer measured using computed tomography, blood test results, and intake of analgesics), subjective reporting of symptom changes, and reports of side effects or death. Ginseng pharmacopuncture improved the objective clinical outcomes in patients with skin wrinkles (width and depth), cervical dysplasia, cancer (in 13 of 18 studies), and amyotrophic lateral sclerosis. The stabilized tumor size in the patients with cancer was reported in two studies. Subjective improvements of symptoms were reported in patients with allergic rhinitis, Behçet’s disease, drug-induced hepatitis, acute demyelinating encephalomyelitis, depression (one RCT), and cancer (11 of 18 studies). Four studies reported the aggravation of diseases such as increase in tumor mass size, metastasis to other organs, and death (one patient) after the treatment session.

#### 3.4.5. Changes in Clinical Outcomes: Examples from Case Reports

**Figure 4:**
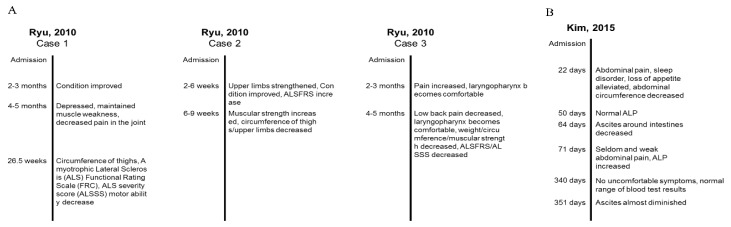
Examples of changes of clinical outcomes induced by ginseng pharmacopuncture over time from two case reports. (**A**) Changes of symptoms of three amyotrophic lateral sclerosis patients after cultivated wild ginseng pharmacopuncture. (**B**) Change of symptoms of a patient with hepatocellular carcinoma after cultivated wild ginseng pharmacopuncture.

To investigate changes in clinical outcomes by treatment session, we summarized two case reports that reported symptoms and clinical indexes over time. Ryu et al. [53] reported three clinical cases of ALS (Figure 4A). In Case 1, combined therapy of three different pharmacopunctures showed the improved general condition after 2–3 months of treatment; however, symptoms and muscular activity were aggravated after 6–7 months of treatment. In Case 2, muscular strength increased after 6–9 weeks of treatment, although the circumference of the limbs decreased. The subject also reported subjective improvement of general condition. In Case 3, pain fluctuated over time, and some symptoms were relieved such as low back pain and discomfort, while muscular strength was decreased. Kim et al. [50] reported a single case of hepatocellular carcinoma, and subjective symptoms (abdominal pain, sleep disorder, and loss of appetite) and ascites were reduced by the combined therapy of wild ginseng pharmacopuncture, acupuncture, and herbal medicine. The alkaline phosphatase (ALP) level returned to normal, and the subject stopped receiving other treatments except medications for hepatitis B (Figure 4B) [50]. In summary, ginseng pharmacopuncture induced improvement of symptoms might vary in each individual and also change over time.

## 4. Discussion

This study provides an overview of the current knowledge of the clinical usage and safety of the use of ginseng pharmacopuncture in place of the traditional oral intake of ginseng. To the best of our knowledge, this is the first review article that covers the clinical usage of ginseng pharmacopuncture by summarizing results of previous clinical studies as well as the toxicity test results in animals and physiological responses in humans. Based on the safety test results in animals, physiological responses to ginseng pharmacopuncture have been conducted, and ginseng pharmacopuncture has been demonstrated to significantly increase the sympathetic nervous system activities and influence protein synthesis in humans. According to the 26 clinical studies, ginseng pharmacopuncture is widely used in clinics, primarily in patients with cancer (lung cancer, hepatocellular carcinoma, prostate cancer, colorectal cancer, pancreatic cancer, cervical cancer, thymus cancer, breast cancer, etc.). In addition to cancer, ginseng pharmacopuncture has been used for the treatment of rhinitis, skin wrinkles, ALS, hepatitis, hepatocirrhosis, and depression, and it has shown significant clinical improvements in patients. Almost all studies applied ginseng pharmacopuncture intravenously, and only a few studies injected it at acupoints.

Previous studies showed the inhibitory effect of ginseng pharmacopuncture on the growth of human non-small cell lung cancer cells (NCI-H460)-induced solid tumor [57] and on the inflammation-related cytokine levels in hepatic metastatic mice model using colon carcinoma cells [58]. The results suggest that ginseng pharmacopuncture could be used in patients with cancer in combination with conventional therapies for cancer (e.g., chemotherapy) by improving the quality of life and general conditions and reducing the AEs of conventional therapies. However, the underlying mechanisms, safety, and clinical efficacy of ginseng pharmacopuncture remain controversial, which necessitates obtaining empirical data from large-scale and well-designed clinical studies.

Although the present review showed that clinical and basic research on ginseng pharmacopuncture has been carried out on many diseases, but it is still far from sufficient. As with other therapies, ginseng pharmacopuncture needs more evidence of its underlying mechanisms and safety at a fundamental level, and requires better evidence of its clinical efficacy and effectiveness from large-scale clinical studies. For example, whether the marker substances, ginsenosides (saponins), of ginseng pharmacopuncture depend on the manufacturing process is controversial. A standard method for extracting the active ingredients of ginseng for pharmacopuncture solution has not been developed, and the extracted substances vary depending on the extraction process (e.g., no index compounds were extracted in the distilled extract of ginseng while ethanol extraction successfully extracted the ginsenosides Rg1 and Rb1 [12]). Baek et al. [59] tested the marker substances of a combined ginseng pharmacopuncture solution comprising distilled extract and alcohol-extracted liquid using high-performance liquid chromatography. The combination method extracted all marker substances (ginsenosides Rg1, Rb1, and Rg3) and did not show significant toxicological changes in rats [59]. However, the clinical effects of ginseng pharmacopuncture manufactured using the combined extraction method (mixture of water-distilled solution and alcohol-extracted compound) have not been studied, while the water-distilled extracts have been mainly tested in our included clinical trials.

As only one RCT using ginseng polysaccharide extract as a pharmacopuncture solution has been reported [16], the clinical effect and safety of ginseng pharmacopuncture described in this review can be interpreted as the complex effects of multiple components in the whole-plant extracts, instead of a single compound. Studies on the pharmacokinetics of active components such as ginsenosides, polysaccharides, fatty acids, essential oils, and phenolic compounds and interactions between multiple components (e.g., a synergistic or antagonistic effect) of ginseng solutions for pharmacopuncture are necessary to demonstrate the pharmacological mechanisms of ginseng pharmacopuncture and develop the most effective solution in the future.

Moreover, among the 26 clinical trials, 15 used intravenous injections and 10 used the acupoint injection method. However, the pharmacokinetics of ginseng pharmacopuncture injected at different injection sites remain unclear. Future studies on various extraction and injection methods in terms of safety, mechanisms, and clinical effects will allow us to improve the efficacy and safety of ginseng pharmacopuncture therapy.

Although not included in this review, the ginseng-related pharmacopuncture research conducted in China often used ginseng extract in combination with various herbs. However, the different types of ginseng pharmacopuncture solution have never been compared to their clinical efficacy and safety. Therefore, clinical studies are necessary to test the effects and toxicity of the many different ginseng extraction methods and injection procedures (intravenous, intramuscular, and acupoints injection), combination of extracts from various herbs as compared with single ginseng extraction, and substance extracted solution as compared with whole plant extracted solution. Future studies should begin with testing the pharmacokinetics and pharmacological interactions of each substance in the ginseng pharmacopuncture solution and compare the substances and effects of ginseng solutions made by various extraction processes. In addition, various toxicity tests in animals and humans should be conducted. These approaches will allow us to find the best manufacturing method to produce the safest and most effective ginseng pharmacopuncture solution. The final and key challenges in the future application of the ginseng pharmacopuncture technique are proving its safety by various toxicity tests such as gene toxicity, carcinogenicity, developmental toxicity, toxicity of single or repeated administration of various doses, and efficacy in patients through a large-scale RCT with an appropriate placebo control. Although ginseng pharmacopuncture has been widely used in TKM clinics, we strongly argue that more concrete evidence is necessary for a safer and more prevalent use of the therapeutic modality in the future. Moreover, quality checks, management of the manufacturing facility, and relevant regulations are required, as pharmacopuncture techniques basically use direct intravenous or intramuscular injection of solutions in the human body.

## 5. Conclusions

In conclusion, although clinical trials and animal experiments showed that ginseng pharmacopuncture treatment is safe and effective for various diseases such as cancer, a higher level of evidence is still needed to confirm its safety and effects before using it widely in the clinical setting. Considering that injection of pharmacopuncture solution could be an alternative to the oral intake of herbal decoction in patients in a coma state or patients with liver damage, we believe that further studies on ginseng pharmacopuncture will bring benefits.

## Figures and Tables

**Table 1:** Chemical constituents of Korean ginseng.

Groups	Contents	Ingredients
Saponin	Saponin (3–6%)	- PPD ginsenosides - PPT ginsenosides - Oleanane ginsenosides
Non-saponin	N-containing substances (12–15%)	- Proteins, amino acids - Peptides, nucleic acids - Alkaloids
	Fat-soluble components (1–2%)	- Fat, fatty acids - Essential oils - Phytosterol - Organic acids - Phenolics - Polyacetylenes - Terpenes
	Carbohydrates (50–60%)	- Polysaccharides - Oligosaccharides - Sugar, fiber, pectin
Others	Ash (4–6%)	- Minerals
	Vitamin (0.05%)	- Water-soluble vitamins

**Table 2:** Chemical structures of protopanaxadiol saponins.

	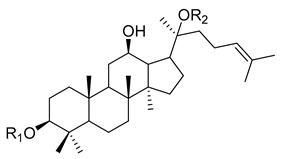	
Ginsenoside	R1	R2
ginsenoside-Ra1	- glc(2→1)glc	- glc(6→1)arap(4→1)xyl
ginsenoside-Ra2	- glc(2→1)glc	- glc(6→1)araf(2→1)xyl
ginsenoside-Ra3	- glc(2→1)glc	- glc(6→1)glc(3→1)xyl
ginsenoside-Rb1	- glc(2→1)glc	- glc(6→1)glc
ginsenoside-Rb2	- glc(2→1)glc	- glc(6→1)arap
ginsenoside-Rb3	- glc(2→1)glc	- glc(6→1)xyl
ginsenoside-Rc	- glc(2→1)glc	- glc(6→1)araf
ginsenoside-Rd	- glc(2→1)glc	- glc
ginsenoside-Rg3	- glc(2→1)glc	- H
ginsenoside-F2	- glc	- glc
ginsenoside-Rh2	- glc	- H
ginsenoside-R1	- glc(2→1)glc(6)Ac	- glc(6→1)glc
ginsenoside-Rs1	- glc(2→1)glc(6)Ac	- glc(6→1)arap
ginsenoside-Rs2	- glc(2→1)glc(6)Ac	- glc(6→1)araf
ginsenoside-Rs3	- glc(2→1)glc(6)Ac	- H
ginsenoside-Rb1	- glc(2→1)glc(6)Ma	- glc(6→1)glc
ginsenoside-Rb2	- glc(2→1)glc(6)Ma	- glc(6→1)arap
ginsenoside-Rc	- glc(2→1)glc(6)Ma	- glc(6→1)araf
ginsenoside-Rd	- glc(2→1)glc(6)Ma	- glc
ginsenoside-R4	- glc(2→1)glc	- glc(6→1)glc(6→1)xyl
ginsenoside-Fa	- glc(2→1)glc(2→1)xyl	- glc(6→1)glc
ginsenoside-X VII	- glc	- glc(6→1)glc

glc: β-d-glucopyranosyl; arap: α-l-arabinopyranosyl; araf: α-l-arabinofuranosyl; xyl: β-d-xylopyranosyl; Ac: acetyl; Ma: malonyl.

**Table 3:** Chemical structures of protopanaxatriol type saponins.

	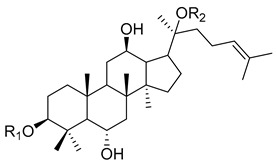	
Ginsenoside	R1	R2
ginsenoside-Re	- glc(2→1)rha	- glc
ginsenoside-Rf	- glc(2→1)glc	- H
20-gluco-ginsenoside-Rf	- glc(2→1)glc	- glc
ginsenoside-Rg1	- glc	- glc
ginsenoside-Rg2	- glc(2→1)rha	- H
ginsenoside-Rh1	- glc	- H
ginsenoside-F1	- H	- glc
ginsenoside-F3	- H	- glc(6→1)arap
ginsenoside-F5	- H	- glc(6→1)araf
ginsenoside-R1	- glc(2→1)xyl	- glc
ginsenoside-R2	- glc(2→1)xyl	- H
ginsenoside-R3	- glc –glc(6→1)	- glc
ginsenoside-R6	- glc –glc(6→1)	- glc*

rha: α-l-rhamnopyranosyl; glc: β-d-glucopyranosyl; arap: α-l-arabinopyranosyl; xyl: β-d-xylopyranosyl; glc*: α-d-glucopyranosyl; araf: α-l-arabinofuranosyl.

**Table 4:** Summary of included clinical trials using (wild) ginseng pharmacopuncture in patients with various diseases.

No. Author (Year), Article Type	Disorder (Symptoms), n *, Gender, Age	Treatment (1, 2, 3…: Serial Treatment or Group; a, b, c…: Combined Treatment)				Significant Results
		Pharmacopuncture		Co-Interventions		
		Herb, Location, Dose, Injection Methods	Duration, Number	Interventions	Duration, Number	
**Case report/series in cancer patients**						
1. Kwon (2005) [34]	Hepatocellular carcinoma, liver cirrhosis, hepatitis B+, lung metastasis (abdominal discomfort), m, 41	a. Cultivated wild ginseng, 0.5–1 cc, IV (at the points of BL13, BL18, LR14, CV12) total 4–40 cc	5 months, 5 times/week	b. Moxibustion, CV4, CV6 c. Cupping	-	CT: near elimination of the cancer cells metastasized into lungs
2. Park (2007) [37]	Squamous cell carcinoma Stage 3B (severe cough, dyspnea, shoulder discomfort), m, 58	2. Cultivated wild ginseng, 10 mL, IV 3. Cultivated wild ginseng, 10 mL, PI (LU1) 4. Cultivated wild ginseng, 10 mL, PI (LU1)	2. 54 days, 1/day 3. 6 days, 1/day 4. 28 days, -	1. Herbal medicine	-	1. CT: aggravation of cancer 2. CT: mass size increased and invaded the mediastinum after the first trial, stabilized after 54 treatments 3. Discontinue the treatment due to a slow of speech and hemiplegia (cerebral infarction) 4. CT: mass invaded the heart Deceased
3. Bang (2008) [38]	Case 1. Lung cancer (adenocarcinoma, Stage T2N3 3B, cough, phlegm), f, 68	a. Cultivated wild ginseng, 20 mL, IV	a. 8 months, 1/day	b. Herbal medication (globule, Hangamdan) c. Herbal medication (granule, decoction)	b. 3/day, 8 months	Overall decrease/maintain of cough and phlegm CT, PET-CT: stabilized mass size
	Case 2. NSCLC stage T1N2 3A, m, 64	Cultivated wild ginseng, IV	a. 5 months	-	-	Overall decrease/maintain of cough, weakness, phlegm CT, PET-CT: stabilized mass size
4. Lee (2010) [39]	Lung cancer (*n* = 3), colorectal cancer (*n* = 2), stomach cancer (*n* = 1), malignant mesothelioma (*n* = 1) (f, 1), median 56	Cultivated wild ginseng, 20 mL, IV	14 days, 1/day (1 cycle)	-	-	median survival days: 544; 1 year survival rate: 57.1%; drop out (*n* = 2), progressive (*n* = 2), stable disease (*n* = 3)
	Case 1: Colon cancer stage 3B, ECOG 3		2 cycles	-	-	Median survival days: 26 days; drop out
	Case 2: Mesothelioma stage 4, ECOG 3		2 cycles	-	-	Median survival days: 56 days; progressive disease; increased size and number of mass
	Case 3: NSCLC stage 4, ECOG 3		1 cycle	-	-	Median survival days: 140 days; drop out
	Case 4: Gastric carcinoma stage 4, ECOG 2, m, 55		13 cycles	-	-	Median survival days: 544 days; stable disease; no changes of stomach mass, increased liver metastasis
	Case 5: Colon cancer stage 4, ECOG 2		3 cycles	-	-	Median survival days: 596 days; progressive disease; increased rectal mass, liver and lung metastasis
	Cace 6: NSCLC stage 3A, ECOG 1, m, 63		13 cycles	-	-	Median survival days: 718 days; stable disease; no changes (early)/increased mass (later)
	Case 7: NSCLC stage 3, ECOG 1, f, 67		21 cycles	-	-	Median survival days: 898 days; stable disease; slight increased mass in left lower lobe (early)/no changes (later), right adrenal gland metastasis (later)
5. Lee (2011) [36]	Prostate cancer, *n* = 2 (f, 0), mean 52	a. Cultivated wild ginseng, 20 mL, IV	-	b. AKDH pharmacopuncture c. Sweet BV pharmacopuncture	-	
	Case 1. Prostate adenocarcinoma (T3bN0M0, fatigue, pain, nocturia, impotence), m, 51	a.	2/week	b. AKDH pharmacopuncture c. Acupuncture	-	Decreased PSA and prostate volume; prostate cancer disappeared, remain cancer in a seminal vesicle and left apex, T3bN0M0 (25 weeks) PSA maintained, no discomfort symptoms (30 months)
	Case 2. Prostate cancer T3bN0M1c (metastasis to bone/lung, pelvic pain, knee pain, short breath, weakness, abnormal urination), m, 53	a.	5/week	c. Sweet BV pharmacopuncture d. Acupuncture e. Medication	2–3/week	Ups and downs of PSA and symptoms, stable health condition (19 months)
6. Kwon (2011) [40]	NSCLC, *n* = 6 (f, 5), median 67	a. Wild ginseng, 20 mL, IV b. Wild ginseng, 10 mL, PI (LU1)	a, b. 4 weeks/cycle			
	Case 1. Adenocarcinoma stage 4, m, 64	2. b 3. a	2. 2 cycles 3. 4 cycles	1. Chemotherapy, operation	-	2. CT: tumor progressed (progressed disease) 3. normal range of blood cancer markers, tumor growth has stopped for 9 weeks, increase of mass (stable disease)
	Case 2. Squamous cell carcinoma stage 4, m, 60	2. a	4 cycles	1. Chemotherapy, operation	-	1. tumor progressed 2. tumor growth has shown stable condition (4 weeks; stable disease); CT: slight progress
	Case 3. Squamous cell carcinoma stage 4, f, 62	b.	2 cycles			CT: Tumor markedly increased (progressed disease)
	Case 4. Breathing difficulties with weight loss, m, 70	b.	1 cycle	-	-	CT: Tumor size slightly increased (stable disease)
	Case 5. Squamous cell carcinoma stage 3A, m, 65	1. a 2. b	1. 5 cycles 2. 2 cycles	-	-	1. CT: tumor growth showed stable condition for 20 weeks (stable disease) 2. CT: tumor size slightly increased (progressed disease)
	Case 6. Squamous cell carcinoma stage 3B (dyspnea, hemoptysis, fever and weight loss), m, 78	a.	16 cycles	-	-	a. all cancer related symptoms and the tumor growth showed stable condition (16 months, stable disease)
7. Kim (2011) [41]	Squamous cell lung carcinoma T2bN1, stage 2B (dyspnea, phlegm, hemoptysis, weight lose), m, 75	a. Cultivated wild ginseng, 0.5 mL, PI (CV12, CV4, BL13)	1/day, 12 days or 2/week, 3 weeks	b. Acupuncture c. Cupping d. Moxibustion		Dyspnea maintained, phlegm and hemoptysis disappeared CT: decrease of mass
8. Im (2012) [42]	Colorectal cancer (metastasis in liver, lung, ovary, chest pain, insomnia), f, 47	a. Cultivated wild ginseng, 10 mL, IV	3–5/week	b. Soram pharmacopuncture ** c. Herbal medicine d. Moxibustion e. Acupuncture	b. 1, 2, 5/day	Decreased pain (2 weeks) Decreased size of metastasized mass in lung (13 weeks) CEA, CA19-9 decreased
9. Ha (2013) [43]	Colorectal adenocarcinoma stage 4B (recurrence, metastasis in liver, spleen, lung), m, 42	a. Cultivated wild ginseng, 30 mL, IV	a. 3/week, 34 weeks	b. FOLFIRI chemotherapy	b. 12/2 weeks, 31 weeks	Recurrence of colorectal adenocarcinoma and metastasis disappeared, no adverse events reported to FOLFIRA chemotherapy
10. Han (2013) [44]	Breast cancer (recurrence, metastasis in lung, chest pain, cough, short breath, shoulder pain, excessive sweating), f, 53	a. Cultivated wild ginseng, 10 mL, IV	3/week	b. Cordyceps militaris pharmacopuncture c. Acupuncture d. Moxibustion e. Herbal medicine	3/week	Chest pain and cough disappeared (1 month) Tubercles in lung disappeared (6 weeks) PET-CT: Recurrence disappeared (50 days)
11. Yun (2013) [46]	Pancreatic cancer (abdominal pain, indigestion, post-prandial pain, abdominal inflation), f, 68	a. Cultivated wild ginseng, 20 mL, NA	3/week, 5 months	b. Medication c. Herbal medication (globule, Hangamdan; globule, Ginseno-pil) d. Acupuncture	b. 4/week c. 3/day	Decreased pain and intake of analgesics (5 months) Improvement of all symptom; CT: decreased hydrothorax and mass size (stable disease)
12. Lee (2013) [47]	NSCLC squamous cell carcinoma stage 4 (cough, dyspnoea, weakness), m, 79	a. Wild ginseng, 10 mL, IV	a. 1/week, 7 months	b. Berbal medication (globule, Soramdan) c. Cordyceps sinensis pharmacopuncture, 10 mL, IV d. Trichosanthes kirilowii pharmacopuncture, 10mL, IV	b. 2/week, 7 months c, d. 1/week, 7 months	ECOG scale maintained (4 weeks) and decreased (5 week) Tumor size decreased
13. Kang (2014) [45]	Case 1. Signet ring cell carcinoma stage 4 (abdominal pain, diarrhea, nausea, fatigue, bloating, heartburn), f, 41	a. Cultivated wild ginseng, 1 mL, subcutaneous injection (EX-B2)	a. 20 times	b. Acupuncture c. Analgesics	b. 46 days, 1/day	Reduced analgesics (10 days) Improvement of symptoms and adverse events of analgesics (sedation, nausea)
	Case 2. bronchioalveolar carcinoma, adenocarcinoma stage 4 (dizziness, nausea, vomiting, insomnia, fatigue, diarrhea, dyspnea), f, 51	a.	a. 5 times	b. Chemotherapy	-	Pain disappeared, able to perform daily activities without analgesics
	Case 3. tubulovillous adenoma, neuroendocrine carcinoma stage 4A (diarrhea, abdominal discomfort, rash, dyspnea), m, 81	a.	a. 13 times	b. Analgesics	-	Decreased pain, improved health condition, reduced analgesics Worsening of liver and kidney functions
14. Park (2015) [48]	Case 1. Thymic cancer (fatigue, fever, anorexia, itching), f, 40	a. Wild ginseng, 1 mL, total 20 mL, subcutaneous injection (EX-B2)	a. 1/2–3 days, 4 times	-	-	Increased white blood cells Decreased erythrocyte sedimentation rate, thyroglobulin Ag level, Korean version of the Revised Piper Fatigue Scale total score
	Case 2. cervical cancer (fatigue, lower limb edema and pain, lower back pain, gait disturbance), f, 61	a. Wild ginseng, 1 mL, total 20 mL, subcutaneous injection (EX-B2)	a. 12 times	-	-	Increased total protein level, lower limb pain Decreased g-glutamyl transferase level, C-reactive protein, fatigue
15. Lee (2015) [35]	Breast cancer stage 3, f, 46	2, 3, 4-a. Wild ginseng, 10 mL, IV	all 3 times/week except 4-e. daily	1. Neoadjuvant chemotherapy, breast conservation surgery, adjuvant radiotherapy 2-b. Cordyceps sinensis 2-c. Trichosanthes kirilowii 2-d. Euonymus alatus 2-e. Astragalus membranaceus pharmacopuncture 3-b. C. sinensis 3-c. T. kirilowii 3-d. E. alatus 3-e. P. vulgaris pharmacopuncture 4-b. E. alatus 4-c. Soram nebulizer solution 4-d. Nebulizer solution 4-e. Herbal medication (globule, Hangamdan)		1. nodule in right upper lung increased 2. 15 mm lymphadenopathies in right paratracheal, interlobar hilar, and subcarinal area 3. lymph node decreased to less than 10 mm 4. no recurrence was observed
16. Kim (2015a) [49]	recurrent oligodendroglioma (hemiparesis, dysarthria, severe daily seizures, headache, drowsiness, constipation, dysuria), m, 54	a. Wild ginseng, 0.5 mL, PI (BL13)	a. 1/week	b. BV pharmacopuncture, GV20, EX-HN1, 0.2 mL c. Acupuncture d. Herbal medicine (fermented red ginseng solution)	c, d. 1/day	brain MRI: decreased tumor size (18 months) free from seizure, left-side hemiparesis was improved, symptoms disappeared (21 months) maintain the treated status without any deterioration (5 years)
17. Kim (2015b) [50]	Hepatocellular carcinoma (stage 2, cirrhosis, abdominal pain, lack of appetite, sleep disorder, fatigue, weakness), m, 57	3-a. Cultivated wild ginseng, 0.25-0.5cc, PI (BL18, CV12, ST25)	3-a. 1/day	1. Ascites puncture, albumin injection, diuretic 2. Ascites puncture 3-b. Medication 3-c. Herbal medication (globule, Hangamdan) 3-d. Acupuncture	various	1. unmanageable liver index and ascites 2. Ascites and symptoms maintained 3. symptoms relieved, abdominal circumference decreased (22 days) normal ALP (50 days) Ascites reduced (64 days) Symptoms disappeared except slight and seldom abdominal pain, increase of ALP/AST (71 days) Symptoms disappeared, normal blood test index (340 days) Ascites almost disappeared (351 days)
18.Lee (2017) [51]	Right breast invasive ductal carcinoma T1N0M0 (metastases in the liver, retroperitoneum, mesentery, pelvic bones, cranium, whole-body bone, pleural effusion, back pain, jaundice, ascites), f, 45	4-a. Wild ginseng, IV or PI (acupoint)	1/day or 2 days	1.Chemotherapy (trastuzumab, paclitaxel) 2. Medication (trastuzumab, vancomycin, tazoperan) 3. Chemotherapy (trastuzumab, paclitaxel) 4-b. Herbal medication (Soramdan) 4-c. Herbal medication (Jeobgoldan)	4-b. 1/day or 3/week 4-c. 2/day	1. - 2. CT, bone scan: reduction of liver metastases, pleural effusion increased, new lesions on the sternum, ribs, acetabulum 3. find vancomycin-resistant enterococci 4-a, b, c. CT: reduction of the liver metastasis lesion, scar, contraction of the liver parenchyma PET-CT: reduction of the tumor size in the right breast, the primary site of the tumor, right axillary lymph node, liver, bone metastases
**Case report/series in patients with other diseases**						
19. Li (1994) [56]	Allergic rhinitis, *n* = 100 (f, 44), range 16-51	Panax ginseng, 4 mL, Sphenopalatine ganglion	1/week, 4–12 week	-	-	Complete symptom relief without recurrence (68%), significant symptom relief (29%), not significant results (3%)
20. Kim (2009) [52]	Case 1. Behçet’s disease (canker sore, edema, chronic fatigue, drug-induced hepatitis), m, 47	a. Cultivated wild ginseng, 10 mL, IV	1–2/week	b. Acupuncture c. AKDH pharmacopuncture	-	Improvement of all symptoms
	Case 2. Drug-induced hepatitis (weakness, fatigue, low back pain, indigestion), m, 51	a. Cultivated wild ginseng, 10 mL, IV	1/week	b. Acupuncture c. AKDH pharmacopuncture	-	Improvement of symptoms, satisfied by the results
	Case 3. Hepatocirrhosis (bleeding, weakness, indigestion), m, 72	Cultivated wild ginseng, 20 mL, IV	3/week	-	-	Improvement of all symptoms, normalization of AST/ALT
21. Ryu (2010) [53]	ALS	a. Cultivated wild ginseng, 20 mL, IV	a. 1–2/2 weeks or 1/2 days	b. AKDH pharmacopuncture c. Sweet BV pharmacopuncture	b. 1–2/2 weeks or 1/2 days	
	Case 1. ALS (myo-atrophy, tetraparesis), f, 51					Improvement of general health condition without muscle weakness (2–3 months) Slow movement, became pessimistic and depressed, slight muscle atrophy (4–5.5 months) Decrease of thigh thickness, ALS functional rating scale, ALS severity score (motor ability) (26.5 weeks)
	Case 2. ALS (myo-atrophy, tetraparesis), m, 47					Increased strength of limbs and ALS functional rating scale (2–6 weeks) Increase of muscular strength, decrease of thickness of thigh/upper limbs and ALS functional rating scale (6–9 weeks)
	Case 3. ALS (myo-atrophy, tetraparesis), f, 70					Increased pain (2–3 months) Decreased low back pain, discomfort (4–5 months) Decreased weight, thickness of body, muscular strength, ALS functional rating scale, ALS severity scale
22. Han (2012) [54]	Cervical dysplasia (genital itching, HPV 52 positive), f, 49	a. Cultivated wild ginseng, 30 mL, IV	6/week, 3 months	b. Herbal medicine c. Moxibustion		Improvement of symptoms, negative HPV 52 test
23. Lim (2014) [14]	Plexiform neurofibroma (general weakness, coldness of the hands/feet, skin rashes), f, 16	2-a. Wild ginseng, 20 mL, IV	1/2 weeks	1. Surgeries 2-b. Sweet BV, 5 mL, intracutaneous	2-b. 1/2 weeks	2. Tumor stopped growing, range of motion improved, no bones or organs affected
24. Park (2014) [55]	Acute demyelinating encephalomyelitis (paraplegia), m, 16	a. Cultivated wild ginseng, 20 mL, IV	1/week, 8 weeks	b. Acupuncture c. Moxibustion d. Herbal medicine e. Rehabilitation f. Medication	b. 2/day c. 1/day	Increased muscular strength, Improved Modified Bathel Index, Normalized muscular motor ability, Decreased pain
25. Lee (2015) [21]	Skin wrinkles, *n* = 23 (f, 20), mean 34	Cultivated wild ginseng, 0.5cc, PI (Ex-HN3, GB1, GB3, LI20, ST4)	2/week, 5 times	-	-	Decreased width and depth of skinfold
**Randomized controlled trial in patients with other diseases**						
26. Liu (2017) [16]	Depression, Intervention group: *n* = 51 (f, 28), range 22-54 Control group: *n* = 51 (f, 29), range 21-56	Intervention group: 1.a. ginseng polysaccharide, 1 mL, PI (BL15, BL20, ST36), 5/week 1.b. Bupleurum pharmacopuncture, 1 mL, PI (BL18, LR8) 1.c. paroxetine	30 days (10 days/session, 3 sessions)	Control group: 2. paroxetine (40–60 mg/day, 1–2 times/day)	-	Intervention group was significantly improved symptoms more than control group at 2, 4, 6 weeks after treatment (The Hamilton Depression Scale (HAMD), *p* < 0.05). Intervention group showed significantly higher total effective rate (98%) than control group (82%). Intervention group reported significantly lower adverse events (7 cases) than control group (21cases).

* Number of samples is omitted in the case of a single subject case study; ** *Astragalus propinquus*, *Curcuma zedoaria* pharmacopuncture. AKDH: ascending kidney water and descending heart fire; ALS: amyotrophic lateral sclerosis; ALT: alanine amino transferase; AST: aspartate transaminase; BL: bladder meridian; BV: bee venom; CT: computed tomography scan; CV: conception vessel meridian; ECOG: Eastern Cooperative Oncology Group scale; EX-B2: extra back 2nd line acupoint; EX-HN: extra head/neck acupoint; f: female; FOLFIRI: chemotherapy made up of folinic acid, fluorouracil, and irinotecan; GB: gallbladder meridian; IV: intravenous injection; n: number of samples; LI: large intestine meridian; LR: liver meridian; LU: lung meridian; MRI: magnetic resonance imaging; NSCLC: non-small-cell lung carcinoma; PET: positron emission tomography; PI: point injection; PSA: prostate specific antigen; ST: stomach meridian.
